# Transmission of Porcine Endogenous Retrovirus Produced from Different Recipient Cells *In Vivo*

**DOI:** 10.1371/journal.pone.0165156

**Published:** 2016-11-10

**Authors:** Nayoung Kim, Jiwon Choi, Sehyun Kim, Yong-Dae Gwon, Yeondong Cho, Jae Myung Yang, Yu-Kyoung Oh, Young bong Kim

**Affiliations:** 1 Department of Life Sciences, Sogang University, Seoul, Republic of Korea; 2 Department of Bio-industrial Technologies, Konkuk University, Neungdong-ro, Gwangjin-gu, Seoul, Republic of Korea; 3 College of Pharmacy and Research Institute of Pharmaceutical Sciences, Seoul National University, Seoul, Republic of Korea; Shanghai Medical College, Fudan University, CHINA

## Abstract

Humanized pigs have been developed to reduce the incidence of immune rejection in xenotransplantation, but significant concerns remain, such as transmission of viral zoonosis. Porcine endogenous retroviruses (PERV), which exist in the genome of pigs, are produced as infectious virions from all porcine cells and cause zoonosis. Here, we examined the possibility of zoonosis of hosts under conditions of immune suppression or xenotransplantation of cells producing host-adapted viruses. Upon transplantation of PERV-producing porcine cells into mice, no transmission of PERV was detected, whereas, transmission of PERV from mice transplanted with mouse-adapted PERV-producing cells was detected. In addition, the frequency of PERV transmission was increased in CsA treated mice transplanted with PERV-producing murine cells, compared with PERV-producing porcine cells. Transmission of PERV to host animals did not affect weight but immune responses, in particular, the number of T cells from PERV-transmitted mice, were notably reduced. The observed risk of PERV zoonosis highlights the requirement for thorough evaluation of viral zoonosis under particular host conditions, such as immunosuppressive treatment and transplantation with host-adapted virus-producing cells.

## Introduction

Xenotransplantation offers the possibility to overcome the shortage of human donor organs [1]. Pigs are preferentially used for xenotransplantation because of ethical considerations, breeding characteristics, compatible organ sizes, and physiology [2, 3]. However, several obstacles, such as immunological barriers and the possibility of viral zoonosis, need to be addressed for successful xenotransplantation [4–6]. To overcome immunological barriers, genetically modified pigs lacking a major xenoantigen have been developed [7, 8]. However, the risk of viral zoonosis remains and is even increased in pigs that are genetically engineered to reduce host-versus-graft reactions [9].

Porcine endogenous retroviruses (PERV) are of considerable interest in the field of xenotransplantation, since the genome is integrated in the germline with a high copy number [10]. To date, PERV-A, PERV-B, PERV-NIH, and PERV-A/C recombinants have been reported to adapt to cell lines via serial passage and infect human cells in vitro [11–15]. No transmission of PERV from various porcine sources to recipient hosts in vivo has been observed [16–19]. However, in exceptional cases, such as immunodeficient animals or non-obese diabetic/severe combined immunodeficiency (NOD/SCID) mouse models, PERV infection and viral gene expression has been detected after transplantation of porcine islet cells [20, 21]. In addition, PERV DNA and RNA have been detected at multiple time-points in human PERV-A receptor 2-expressing transgenic mice, indicating that the virus is able to replicate after xenotransplantation [22]. Although zoonosis is clearly caused by xenotransplantation of pig organs, the frequency of viral transmission from xenotransplant sources in host animals remains to be established. Moreover, little is known about the potential pathological risks in host animals undergoing transient immunosuppressant treatment. Human cell-adapted PERV-A/C was shown to infect cells from non-human primates without selection pressure. This phenomenon was attributed to generation of PERV with changes in the long terminal repeat, which plays an important role in viral replication [23].

In this study, we examined the hypothesis that PERV from different sources xenotransmit, infect, and integrate into genomes of hosts undergoing immunosuppressant treatment. To this end, mouse cells producing murine cell-adapted PERV (mPERV) or porcine cells producing porcine PERV (pPERV) were transplanted into host mice, and transmission of PERV in mice was analyzed under the condition of immunosuppressive treatment. Here, we reported that the viral zoonosis occurred at a higher frequency in BALB/c mice transplanted with mPERV-producing mouse cells than pPERV-producing porcine cells and in the mice under immunosuppressant treatment, relative to untreated condition.

## Materials and Methods

### Animals and cell lines

NIH3T3 and PK15 (American Type Culture Collection, Manassas [ATCC], VA, USA) were cultured in Dulbecco's modified Eagle's medium (Thermo Fisher Scientific, Waltham, MA, USA) supplemented with 10% fetal bovine serum (Thermo Fisher Scientific). Four week-old female BALB/c mice purchased from Orient-Bio (Seungnam, Kyonggi-do, Republic of Korea) and housed in filter-top cages, with water and food provided *ad libitum*. Mice were maintained in accordance with the Guide for the Care and Use of Laboratory Animals of Konkuk University (Seoul, Republic of Korea), and were housed in a Bio-safety Level 2 facility, with implementation of the appropriate biosafety practices. NOG (NOD/Shi-*scid*/IL-2Rγ^null^) mice were purchased from Jackson Laboratory (Bar Harbor, ME, USA) and housed in a SPF facility. The use of animals in these experiments was approved by the Institutional Animal Care and Use Committee of Konkuk University (Approval No. KU14146-1). Cyclosporin A (10 mg/kg/day) (CsA, Chong Kun Dang Pharmaceutical Corp., Seoul, Korea) in olive oil (Sigma-Aldrich, St. Louis, MO, USA) was injected intraperitoneally for immunosuppressive treatment.

### Construction of a PERV-producing NIH3T3 (mPERV/NIH3T3) cell line

The PERV/NIH3T3 cell line was constructed by co-transfection of pcDNA3.1 (Invitrogen, Carlsbad, CA, USA) and the previously reported PERV-B clone [24] into NIH3T3 cells. Cells were cultured in Dulbecco's modified Eagle's medium containing 1.5 mg/ml neomycin using GenePORTER2 (Gene Therapy Systems, CA, USA) and ten clones of neomycin-resistant PERV/NIH3T3 cells were isolated 4 weeks later.

### PCR-based assays of PERV/NIH3T3 cells

Genomic DNA (gDNA) was isolated from 5 PERV/NIH3T3 clones using a DNeasy Blood & Tissue kit (Qiagen, Valencia, CA, USA). PERV pol gene was amplified via nested PCR with 200 ng gDNA and the primers PERVPol1 (GATGAGCGTAAGGGAGTAGC) and PERVPol2 (TGCTTCCGTCAGTGAACCAG) for primary PCR and PERVPol3 (CCATACTGGTCAAGGACG) and PERVPol4 (TCATCAGTCTCTTCAGGC) for secondary PCR.

Viral RNA was extracted from the supernatant of NIH3T3 cells transfected with the PERV-B clone using TRIzol (Thermo Fisher Scientific). RNA was mixed with AccuPower CycleScript RT PreMix (Bioneer, Daejeon, Korea) containing a random 9 mer, and incubated at 42°C for 60 min. Nested PCR was performed with the synthesized cDNA, Premix Ex Taq (Takara Bio, Inc., Otsu, Japan) and previously described primers [24].

To estimate the genomic copy number of PERV, real-time PCR was performed with PERVPol3 and PERVPol4 primers and the RT product as template [24] using the SYBR Green PCR Master Mix Kit (Applied Biosystems, Foster City, CA, USA) in the ABI Prism 5700 sequence detection system (Applied Biosystems). The number of PERV copies per milliliter was determined by extrapolating the standard curve generated using the pol gene cloned into pGEM-T-Easy (Promega, Fitchburg, WI, USA).

### Cell transplantation and detection of the viral genome

The mice were anesthetized with an intramuscular injection of mixture of zoletil and rompun during the entire surgical procedure. NIH3T3, PERV/NIH3T3, or PK15 cells were injected subcutaneously (10^7^ cells/mouse) into mouse abdomen or transplanted into the right kidney via the kidney capsule method [25, 26]. The incised skin area was disinfected with 70% alcohol for three days after transplantation. All mice were subject to post-operative monitoring such as general appearance and locomotor activity twice a day and body weight change once a day. All animals did not exhibit any observable abnormalities except for the subcutaneously transplanted mice which had swelling around the injected area for three days. The mice were sacrificed 6 week after transplantation by the inhalation of carbon dioxide and then each organ was extracted. Transmission of PERV was determined in organs of transplanted mice at both the DNA and RNA levels. Various organs, including brain, liver, lung, heart, left kidney, and spleen, were isolated six weeks after cell transplantation and homogenized with BioMasher. Total DNA and RNA were extracted from 0.2 mg homogenized organ samples using a DNeasy Blood & Tissue kit (Qiagen, Valencia, CA, USA) and an RNeasy kit (Qiagen), respectively. gDNA (200 ng) and RNA (1 μg) were used in nested PCR and RT-PCR, respectively. Murine glyceraldehyde-3- phosphate dehydrogenase (mGAPDH) was used for normalization of gene expression using the primers: 5′-ATCACCATCTTCCAGGAGCGAGA-3′ (sense) and 5′-GCTTCACCACCTTC TTGATGTCA-3′ (antisense). To test for potential microchimerism, the neomycin gene was examined with the specific PCR primers: 5′-ATGATTGAACAAGATGGATTGCAC-3′ (sense) and 5′-CCATGATGGATACT TTCTCGGCAG-3′ (antisense). The porcine mitochondrial DNA cytochrome oxidase subunit II (COII) gene was detected in PK15 cells with the primers: 5′-TCACCCATCATAGAAGAACTCCTACA-3′ (sense) and 5′-TTTTACGGTTAAGG CTGGGTTATTAAT-3′ (antisense).cytochrome oxidase

### PERV infection from sera of NOG (NOD/Shi-scid/IL-2Rγnull) mice transplanted with PERV/NIH3T3 cells and Viral Titration

Six NOG mice were divided into two groups and NIH3T3 and PERV/NIH3T3 cells were injected subcutaneously (10^7^ cells/mouse) in the abdomen of each mouse. After 6 weeks, blood was collected and sera were added to 293T cells in Transwell (Corning, Lowell, MA, USA), with seeding of 293T cells (10^5^ cells/well) in the lower wells. DNA was isolated 7 days after 293T cell treatment using a DNeasy Blood & Tissue kit (Qiagen). For PCR, 200 ng gDNA was used as a template with the following primers: PERVPol1, PERVPol2, PERVPol3, and PERVPol4. Amplified products were cloned into T-vector (T-Blunt PCR Cloning Kit, Solgent, Seoul, Korea) and sequenced.

The viral titer from NOG mouse serum was determined from the copy number of viral RNA. The reverse transcription RNA product isolated from NOG serum was used as the template for real-time PCR for the PERV pol gene using the SYBRGreen PCR Master Mix Kit (Applied Biosystems) with primers nABCPol3 and nABCPol4. Pol gene-specific PCR products were continuously measured using the ABIPrism5700 Sequence Detection System (Applied Biosystems) during the 50 cycles of amplification. The number of PERV copies per ml was determined by the extrapolation of the standard curve generated using pGEM-T-Easy vector (Promega) containing the pol gene insert.

### Detection of PERV proteins by immunohistochemistry

To examine the expression patterns of PERV proteins, organs of transplanted mice were isolated, embedded in paraffin, and sectioned at a thickness of 6 μm. Sections were mounted on positively charged slides, air-dried in a 40°C incubator overnight, and deparaffinized in xylene. After rehydration in graded alcohol, tissues were incubated in 1% hydrogen peroxide diluted with methanol for 15 min to block endogenous peroxidase activity, followed by rehydration in distilled water and phosphate-buffered saline (PBS, pH 7.4). Tissues were incubated in blocking solution (3% bovine serum albumin and 10% normal calf serum in PBS) for 60 min at room temperature, followed by incubation with rabbit anti-PERV env antibody (1:1000) [27] at 4°C overnight. Next, tissues were washed with PBS containing 0.1% Triton X-100, and incubated with biotinylated mouse anti-rabbit antibody (Dako, Glostrup, Denmark) followed by streptavidin conjugated to HRP peroxidase (Dako), and subsequently visualized with 3,3′-diaminobenzidine. Hematoxylin was employed as a nuclear counterstain.

All surgical operations were performed on sterilized dissecting pan. To minimize suffering, mice were sedated by mixture of tiletamine and xylazine anesthesia (50 and 5 mg/kg of body weight, respectively).

### Analysis of B cells and T cells by flow cytometry

Splenocytes were treated with red blood cell lysis solution (Sigma-Aldrich, Inc., St. Louis, MO, USA) at 4°C for 5 min, and washed twice with PBS containing 1% fetal bovine serum. Subsequently, splenocytes were incubated with FITC-conjugated hamster anti-mouse CD3e (CD3ε chain) monoclonal antibody (BD Biosciences, San Jose, CA, USA), followed by PE-conjugated rat anti-mouse CD45R/B220 monoclonal antibody (BD Biosciences). To measure the proportions of CD4+ and CD8+ T cells in splenocytes, cells were incubated with PE-conjugated rat anti-mouse CD4 (L3T4) monoclonal antibody (BD Biosciences) and FITC-conjugated rat anti-mouse CD8 monoclonal antibody (BD Biosciences) for 30 min at 4°C. Cells were washed twice with PBS, resuspended in 1 ml PBS, and analyzed using a FACSCalibur flow cytometer (Becton Dickinson, Franklin Lakes, NJ, USA).

### Statistical analysis

The control and treatment groups were compared using the unpaired Student’s t-test. A p value <0.05 was considered significant.

## Results

### Construction of the mPERV-producing murine cell line, PERV/NIH3T3 and confirmation of PERV

**Fig 1:**
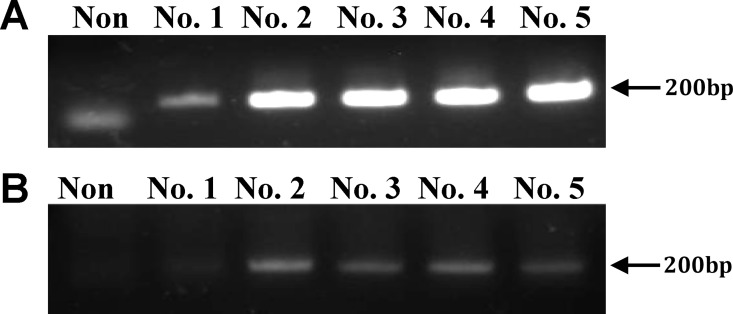
Detection of PERV genes expressed from PERV/NIH3T3 cells by PCR. (A) PCR products of PERV pol gene by nested PCR from gDNA isolated from five cell lines (Nos. 1–5) of PERV/NIH3T3. (B) RT-PCR products of the PERV pol gene from the supernatants of five PERV/NIH3T3 cell lines. Lane Non, non-transfected NIH3T3 cells. Arrows indicate the expected size of amplified pol.

To mimic possible viral zoonosis by a host species-adapted virus, murine cell-adapted PERV was used as an experimental virus for mouse infection. Murine cells producing mPERV (mPERV/NIH3T3) were generated by transfection with the PERV genome along with the plasmid containing a neomycin-resistant gene for transfection selection. Nested PCR led to the identification of five neomycin-resistant cell lines (Nos. 1–5) in which the PERV gene was integrated (Fig 1A), among which four (Nos. 2–5) were validated as PERV-releasing cell lines by RT-PCR of the supernatant (Fig 1B). The highest titer among virus-releasing cells (1.8 × 10^6^ particles/mL virus) was detected from the supernatant of cell line No. 2. Accordingly, PERV/NIH3T3 No. 2 cells were utilized as mouse donor cells for transplantation in subsequent experiments.

### Detection of the PERV gene in mice transplanted with PERV-producing cells

**Fig 2:**
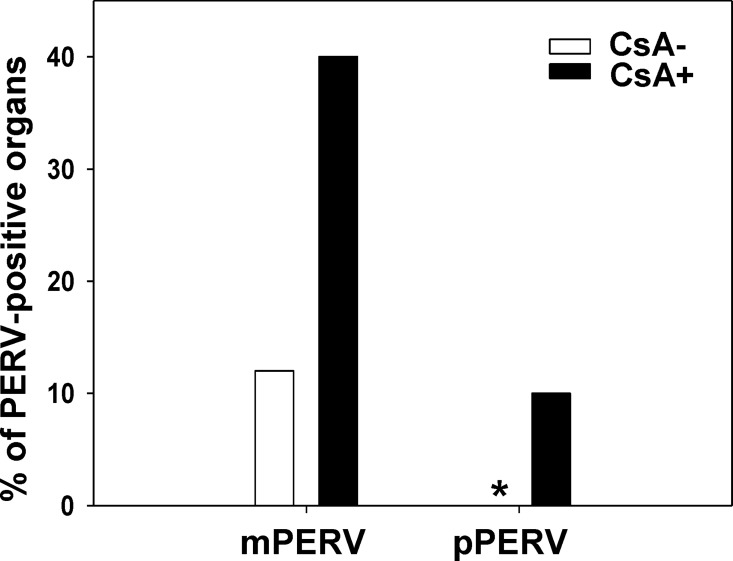
Percentage of PERV-positive organs from mice transplanted with PERV/NIH3T3 or PK15 in the presence or absence of CsA. PERV-positive organs were analyzed based on genomic DNA from individual organs of mice transplanted with PERV/NIH3T3 (mPERV) and PK15 (pPERV) by PCR. *: PERV was not detected in all organs.

To determine whether transmission of PERV is affected by the origin of the virus in vivo, host-adapted PERV/NIH3T3 cells were transplanted and compared with cells in which PERV was originally detected (pPERV-producing PK15). Mice were further analyzed to determine the effects of cyclosporin A (CsA) as well as transplant route, specifically, subcutaneous injection (S.C.) and kidney capsule (K.C.), on zoonosis efficiency. Six organs (brain, liver, lung, heart kidney, spleen) from 5 and 6 mice in the S.C. and K.C. transplant groups were examined under CsA and no treatment conditions. No xenotransmission by pPERV was observed in the absence of CsA. In contrast, PERV-positive organs were detected following transplantation of mice with PERV/NIH3T3 with no CsA treatment, as shown in Fig 2. Upon treatment of mice with CsA, the percentage of PERV-positive organs in the mPERV transmission group was about 3.5 times higher than that in the pPERV group. The percentage of PERV-positive organs from mice treated with CsA was higher, compared to untreated mice. In mice transplanted with PERV-releasing cells via either subcutaneous injection or the kidney capsule method, similar percentage of PERV-positive organs was detected regardless of the origin of PERV (Table 1). The percentage of PERV-positive organs shown in Fig 2 and Table 1 was calculated based on PCR-based gDNA evaluation, and PERV RNA data obtained using RT-PCR supported the finding that the route of transplantation into mice do not affect the incidence of xenotransmission (Table 2). Detection of PERV-positive organs in xenotransplanted mice under the above specific conditions surprisingly revealed that transplantation of donor cells in which PERV proviral genes were integrated resulted in zoonosis. A further unexpected result is that donor cells releasing host-adapted virus have critical effects on the incidence of zoonosis.

**Table 1:** Percentages of PERV-positive organs from mice transplanted with PERV/NIH3T3 or PK15 with no or CsA treatment.

	Percentage of PERV-positive organs			
Cells	CsA+		CsA-	
	S.C.	K.C.	S.C.	K.C.
**NIH3T3**	0/30 (5) 0%	0/30 (5) 0%	0/30 (5) 0%	0/30 (5) 0%
	0/60, **0%**		0/60, **0%**	
**PERV/NIH3T3**	10/30 (5) 33%	16/36 (6) 44%	3/30 (5) 10%	3/24 (4) 13%
	26/66, **39%**		6/54, **11%**	
**PK15**	3/30 (5) 10%	3/30 (5) 10%	0/60 (10) 0%	N/A
	6/60, **10%**		0/60, **0%**	N/A

PCR-based gDNA detection was performed in six organs (brain, liver, lung, heart, kidney, spleen) from the number of mice shown in brackets. S. C., subcutaneous injection; K.C., kidney capsule method.

**Table 2:** PERV-positive organs in infected mice.

CsA treated group	Organs	PERV-positive (gDNA)^a^	PERV-positive (RNA)^b^	PERV-infected mice^c^	CsA untreated group	Organs	PERV-positive (gDNA)^a^	PERV-positive (RNA)^b^	PERV-infected mice^c^
**G1** (NIH3T3)S.C.	Brain	0/5	0/5	0/5	**G 7** (NIH3T3) S.C.	Brain	0/5	0/5	0/5
	Liver	0/5	0/5			Liver	0/5	0/5	
	Lung	0/5	0/5			Lung	0/5	0/5	
	Heart	0/5	0/5			Heart	0/5	0/5	
	Kidney	0/5	0/5			Kidney	0/5	0/5	
	Spleen	0/5	0/5			Spleen	0/5	0/5	
**G2** (NIH3T3)K.C.	Brain	0/5	0/5	0/5	**G8** (NIH3T3) K.C.	Brain	0/5	0/5	0/5
	Liver	0/5	0/5			Liver	0/5	0/5	
	Lung	0/5	0/5			Lung	0/5	0/5	
	Heart	0/5	0/5			Heart	0/5	0/5	
	Kidney	0/5	0/5			Kidney	0/5	0/5	
	Spleen	0/5	0/5			Spleen	0/5	0/5	
**G3** (PERV/NIH3T3) S.C.	Brain	0/5	1/5	4/5	**G9** (PERV/NIH3T3) S.C.	Brain	0/5	0/5	2/5
	Liver	2/5	1/5			Liver	1/5	0/5	
	Lung	1/5	2/5			Lung	0/5	0/5	
	Heart	1/5	1/5			Heart	1/5	0/5	
	Kidney	3/5	2/5			Kidney	0/5	0/5	
	Spleen	3/5	1/5			Spleen	1/5	1/5	
**G4** (PERV/NIH3T3) K.C.	Brain	3/6	1/6	5/6	**G10** (PERV/NIH3T3) K.C.	Brain	0/4	0/4	3/4
	Liver	3/6	1/6			Liver	3/4	0/4	
	Lung	0/6	2/6			Lung	0/4	1/4	
	Heart	2/6	2/6			Heart	0/4	0/4	
	Kidney	4/6	1/6			Kidney	0/4	0/4	
	Spleen	4/6	2/6			Spleen	0/4	0/4	
**G5** (PK15) S.C.	Brain	0/5	1/5	2/5	**G11** (PK15) S.C.	Brain	0/10	0/10	0/10
	Liver	1/5	0/5			Liver	0/10	1/10	
	Lung	0/5	0/5			Lung	0/10	0/10	
	Heart	1/5	0/5			Heart	0/10	0/10	
	Kidney	0/5	0/5			Kidney	0/10	0/10	
	Spleen	1/5	1/5			Spleen	0/10	0/10	
**G6** (PK15) K.C.	Brain	1/5	0/5	2/5					
	Liver	2/5	1/5						
	Lung	0/5	0/5						
	Heart	0/5	0/5						
	Kidney	0/5	0/5						
	Spleen	0/5	0/5						

^a^ PCR results with 200 ng gDNA (number of PCR-positive organs/number of total organs).

^b^ RT-PCR results with 1 μg total RNA (number of RT-PCR-positive organs/number of total organs).

^c^ PERV-positive mice evaluated with PCR using gDNA (number of PERV-positive mice/number of total mice).

### Confirmation of PERV zoonosis by detection of PERV env protein from the spleen

**Fig 3:**
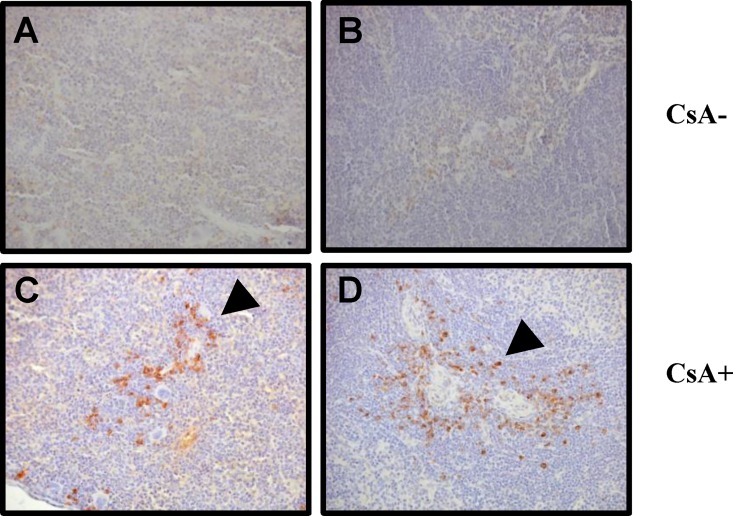
Immunohistochemical detection of PERV env protein from spleen of PERV-infected mice. Immunohistochemistry was performed in spleens from CsA-non treated mice transplanted with NIH3T3 cells (A), non-treated mice transplanted with PERV/NIH3T3 cells (B), CsA-treated mice transplanted with PERV/NIH3T3 cells by subcutaneous injection (C), and CsA-treated mice transplanted with PERV/NIH3T3 cells by the kidney capsule method (D). Arrowheads in (C) and (D) indicate staining of PERV env protein as brown spots.

PCR-based detection of the PERV gene from genomic DNA in transplanted mice was confirmed using immunohistochemical analysis in spleens of mice transplanted with PERV/NIH3T3. As shown in Fig 3, brown spots centralized in lymphatic nodules composed of the white pulp of spleen were detected in CsA-treated mice transplanted with PERV/NIH3T3 via both subcutaneous injection and the kidney capsule method. Although the PERV pol gene was effectively detected by PCR regardless of CsA treatment, PERV env was only detected in mice transplanted with PERV/NIH3T3 cells under CsA treatment using immunohistochemistry (Fig 3). PERV env detection specifically from CsA-treated mice may be attributed to high expression of the protein, which is consistent with PERV DNA detection results showing a 3.5 times higher percentage of PERV positivity in CsA-treated than non-treated mice. PCR and immunohistochemistry data collectively suggest that PERV is xenotransmitted from donor cells to host animals, and immunosuppressive CsA treatment increases the possibility of PERV zoonosis.

### Infection of NOG mice with PERV from transplanted mice

Significant involvement of the immune system in the higher incidence of PERV xenotransmission was further confirmed in NOG (NOD/Shi-scid/IL-2Rγnull) mice transplanted with PERV-producing PERV/NIH3T3 cells. Severely immunodeficient NOG mice exhibit disrupted T, B and NK cell development as well as reduced innate immunity. Accordingly, PERV xenotransplantation was tested in NOG mice to mimic the immunosuppressed condition of normal mice via CsA treatment. In addition to analysis of PERV xenotransmission in NOG mice, the potential route of infection by the virus released from transplanted mouse blood was assayed by examining infection of 293T cells cultured in the lower well of a transwell. Sera from transplanted NOG mice were placed in the upper well of transwell. Physical separation using a membrane between sera and 293T cells aimed to exclude possible false-positive PERV detection due to microchimerism. Genomic DNA isolated from 293T cells was positive for PERV pol, as determined using PCR. The sequence of the pol region was identical to the pol gene of PERV-B (EU523109) incorporated in PERV/NIH3T3 cells S1 Fig. This result confirmed that recipients of PERV-producing cells can be infected through blood from transplanted mice and not contaminant donor cells.

### Exclusion of the possibility of microchimerism from donor cells in host

**Fig 4:**
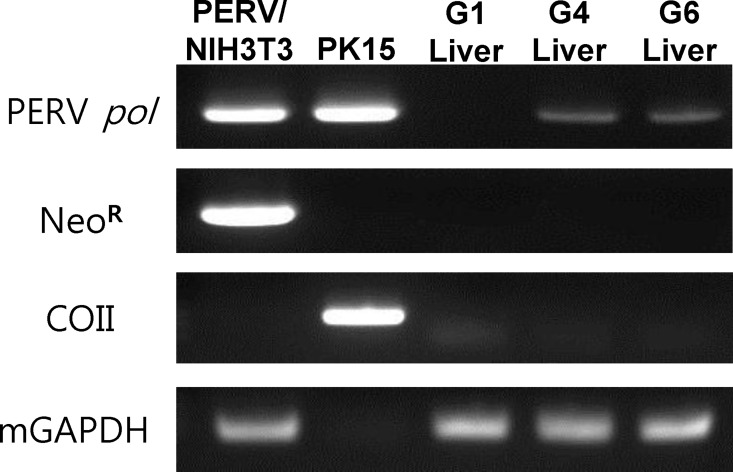
PCR detection of PERV pol, neomycin-resistant, and porcine mitochondrial COII genes in mice transplanted with PERV/NIH3T3 or PK15 cells. PERV pol (200bp), neomycin-resistant (300bp), porcine mitochondrial COII (250bp), and murine GAPDH (100bp) genes were detected from gDNA extracted from PERV/NIH3T3 cells, PK15 cells, and liver tissues from Group 1 (G1), Group 4 (G4), and group 6 (G6).

To exclude the possibility of microchimerism (presence of cellular DNA from transplanted cells), PCR was performed to detect PERV pol, neomycin-resistant gene, porcine mitochondrial COII gene, and murine GAPDH. As shown in Fig 4, G4 and G6 mice transplanted with PERV/NIH3T3 and PK15 cells, respectively, were positive for PERV pol DNA, while G1 mice transplanted with NIH3T3 cells tested negative. G4 and G6 mice were negative for the marker gene of PERV donor cells, neomycin-resistant gene, and porcine mitochondrial COII, respectively (Fig 4). Our findings indicate that PERV positivity in organs results from zoonosis from PERV/NIH3T3 and PK15 cells not from the contaminated DNAs from transplanted cells.

### Weight changes of PERV-xenotransmitted mice under CsA treatment

**Fig 5:**
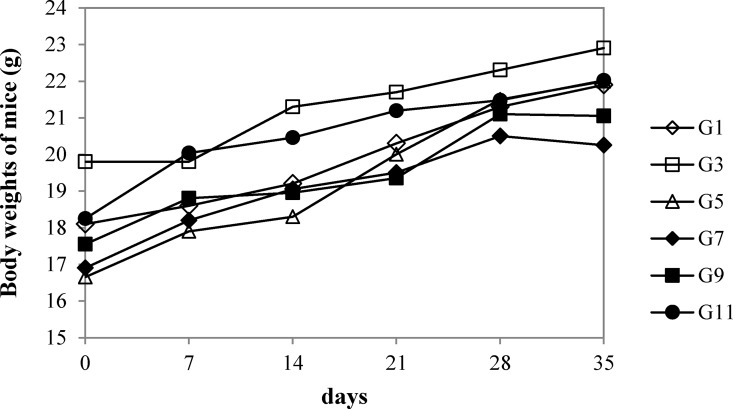
Body weights of PERV-infected or non-infected mice after CsA treatment. Weights of animals in each group were measured every week for 5 weeks. Each dot indicates the body weight of mice in CsA-treated (G1, G3, G5), untreated (G7, G9, G11), PERV-infected (G3, G5, G9) or uninfected groups (G1, G7, G11).

To determine whether xenotransmitted PERV affects pathogenicity in recipient mice, weight changes were monitored for 5 weeks between xenotransmitted (G3, G5, and G9; Table 2) and non-transmitted groups (G1, G7, and G11; Table 2) identified based on PERV positivity in their genomic DNA. Mice transplanted with PERV-producing cells using the kidney capsule method were excluded for comparison purposes because the procedure involved surgery. As shown in Fig 5, no notable weight changes in mice were observed between the two groups. In addition, the origin of PERV and CsA treatment of transplanted mice had no effect on weight.

### Effect of PERV infection on immune response

**Fig 6:**
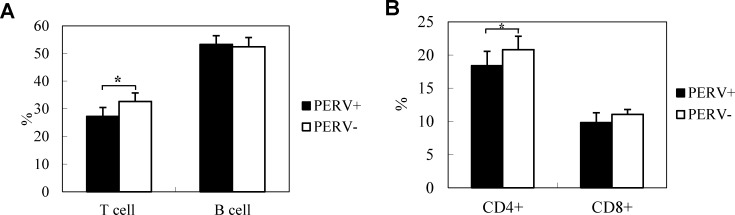
Effects of PERV infection on lymphocytes and CD4/8 cells in host animals. Cells in splenocytes isolated at 7 weeks post-transplantation were stained with fluorescence-conjugated antibodies, and the number of labeled cells were measured by FACS. (A) Percentage of T and B cells in splenocytes of CsA-treated (n = 24) and untreated (n = 20) mice. (B) Percentage of CD4+ and CD8+ T cells in splenocytes of PERV-infected (n = 6) and uninfected (n = 6) mice. *: significantly different between PERV-infected and non-infected groups (Student t-test, p<0.05). The error bars indicate standard deviations.

Immunological responses were compared between PERV-positive and -negative animals specifically in the CsA-treated group. As shown in Fig 6A, the percentage of T cells isolated from spleens of PERV-positive mice was 5.4% lower, compared with that in the PERV-negative mice, while the percentage of B cells was not significantly affected by PERV infection. In particular, the CD4-positive T cell number in PERV-positive mice was significantly reduced, compared with the PERV-negative group whereas CD8-positive T cells were only marginally reduced, as determined using the student's t-test (Fig 6B).

## Discussion

To maximize the potential utility of pig organs as a source of xenotransplantation, extensive research is required to avoid virus-contaminated organs as donors for the effective prevention of zoonosis and related complications. Since successful xenotransplantation is usually performed under immunosuppressive conditions to minimize the risk of immune rejection, the susceptibility of hosts under diverse conditions, such as immunosuppressant treatment or infection by a host-adapted virus, needs to be assessed. PERV transmission has not been reported after transplantation of porcine organ/islets or PERV-producing cells. However, when the host was transplanted with cells producing host-adapted virus, PERV transmission was observed in our experiments. Moreover, the zoonosis rate was increased upon treatment of the host with immunosuppressant.

Transmission of PERV in the organs of host animals was analyzed via PCR detection of the PERV pol gene. The possibility of misleading results on PERV detection owing to contamination of transplanted cells was discounted based on negative PCR results for neomycin or porcine mitochondrial COII gene in PERV-positive organs. Following incubation of sera from mice transplanted with PERV/NIH3T3 cells on top of a transwell membrane, detection of PERV in 293T cells at the bottom provided evidence that the virus particles migrate through the membrane and infect cells. Additionally, detection of PERV in brain samples excluded the possibility of contamination. Transplanted cells cannot migrate to the brain due to the blood-brain barrier (BBB); therefore, the detected PERV gene likely originated from transmission of virus particles, rather than donor cell contamination. The mechanism by which human immunodeficiency virus (HIV) disrupts BBB and facilitates entry into brain, resulting in NeuroAIDS has been extensively studied. Specifically, HIV infection affects constituent proteins associated with the gap junction and astrocytes in BBB [28, 29]. The source of PERV RNA and mechanism of crossing BBB are currently under investigation. A possible explanation for detection of PERV RNA in brain might be derived from the mechanism of HIV that viral infection alters BBB integrity, enabling virus entry into the brain.

Previously, mouse retrovirus-mediated PERV transmission was demonstrated in mice xenografted with human cells [30]. Transmitted PERV was identified as a retroviral pseudotyped virus, and pseudotyping between PERV and murine leukemia virus (MLV) reported as a well-established phenomenon. In addition, xenotropic MLV (MLV-X) was shown to be activated by immunosuppression, and efficiently expanded the tropism of all known PERV classes [31]. Detection of PERV in transplanted animals in this study excluded the possibility of MLV-X pseudotyped PERV demonstrated earlier, since the NIH3T3 cells used were non- permissive to MLV-X [31]. More importantly, mPERV was more infectious than pPERV, highlighting the importance of viral envelope origin and further implying that the envelope protein is not from the same source, MLV-X, but from the cell line-adapted PERV envelope. A previous study reported the presence of mERV-XL in NIH3T3 cells [32]. Accordingly, the possibility of generation of recombinant PERV with the left half of the XMRV was examined and eventually discounted based on sequencing of the pol gene of mPERV as well as the absence of PCR products with primers specific for the left half of the mERV-XL genome and the right half of the PERV genome S2 Fig. The maintenance of the structure of PERV env, detected via immunohistochemistry using a specific PERV env antibody, provided further evidence that the virus from PERV/NIH3T3 is host-adapted and not recombinant PERV. This finding provides an important clinical consideration when humanized porcine organs are transplanted in humans. Furthermore, as reported for PERV viremia in pigs [33], PERV from humanized pig organs may systemically spread to various organs, which explains the result of PERV spreading to the spleen and nontransplanted left kidney.

Notably, higher transmission of mPERV in host animals under immunosuppressant treatment may be, in part, due to the enhanced likelihood of immune escape of the transplanted virus. CsA is generally used as an immune suppressant to reduce immune rejection in organ transplantation, since the major histocompatibility complex (MHC) classes of cells used for transplantation are different from those of host animals [34, 35]. Thus, the immune escape virus may be critical for zoonosis in the host when the immune system is suppressed for transplantation. In addition to low immune state, PERV infection affected T lymphocytes, resulting in a more suppressed immune system in host, although the underlying mechanism requires elucidation.

In conclusion, data from this study have provided insights into the conditions and mechanisms promoting zoonosis in xenotransplantation. To achieve safe xenotransplantation, further research focus on the routes of PERV trafficking is required, with a view to developing techniques for repression, inactivation or blockage of transmission.

## Supporting Information

S1 File NC3Rs ARRIVE Guidelines checklist. (PDF)

S1 Fig The sequence alignment of PERV molecular clone B (EU23109) with PCR product. The genomic DNA was isolated from 293T cells treated with serum from NOG mice transplanted with PERV/NIH3T3 cells. Nested PCR product using pol1, pol2, pol3, and pol4 as primers was cloned to pGEM-T Easy vector and the pol sequence was compared with PERV molecular clone B (EU23109).
(PPTX)

S2 Fig Detection of PCR products from the genomic DNA isolated from PERV/NIH3T3 cells. (A) The expected annealing location of primers (arrows) was illustrated in the genomes of PERV and mERV-XL (32). (B) The PCR products of PERV gag-pol and mERV-XL gag-pol with the listed primers were detected in lanes 1, 6 and lanes 2, 3, respectively. The PCR product of a possible recombinant PERV with mERV-XL was not detected in lane 4,5,7,8. (C) Nucleotide sequence of the primers used for the PCR in (B) was listed.
(PPTX)

## References

[1] Dorling A, Riesbeck K, Warrens A, Lechler R. Clinical xenotransplantation of solid organs. Lancet. 1997;349(9055):867–71. Epub 1997/03/22. 10.1016/S0140-6736(96)09404-4 . 9121275

[2] Bach FH. Xenotransplantation: problems and prospects. Annual review of medicine. 1998;49:301–10. Epub 1998/03/24. 10.1146/annurev.med.49.1.301 . 9509265

[3] Cooper DK, Keogh AM, Brink J, Corris PA, Klepetko W, Pierson RN, et al Report of the Xenotransplantation Advisory Committee of the International Society for Heart and Lung Transplantation: the present status of xenotransplantation and its potential role in the treatment of end-stage cardiac and pulmonary diseases. The Journal of heart and lung transplantation: the official publication of the International Society for Heart Transplantation. 2000;19(12):1125–65. Epub 2000/12/22. . 11124485 10.1016/s1053-2498(00)00224-2

[4] Boneva RS, Folks TM, Chapman LE. Infectious disease issues in xenotransplantation. Clinical microbiology reviews. 2001;14(1):1–14. Epub 2001/01/09. 10.1128/CMR.14.1.1-14.2001 11148000 PMC88959

[5] Gunzburg WH, Salmons B. Xenotransplantation: is the risk of viral infection as great as we thought? Molecular medicine today. 2000;6(5):199–208. Epub 2000/04/27. . 10782067 10.1016/s1357-4310(00)01708-1

[6] Samstein B, Platt JL. Physiologic and immunologic hurdles to xenotransplantation. Journal of the American Society of Nephrology: JASN. 2001;12(1):182–93. Epub 2001/01/03. . 11134266 10.1681/ASN.V121182

[7] Lai L, Kolber-Simonds D, Park KW, Cheong HT, Greenstein JL, Im GS, et al Production of alpha-1,3-galactosyltransferase knockout pigs by nuclear transfer cloning. Science. 2002;295(5557):1089–92. Epub 2002/01/05. 10.1126/science.1068228 . 11778012

[8] Dufrane D, Gianello P. Pig islet for xenotransplantation in human: structural and physiological compatibility for human clinical application. Transplant Rev (Orlando). 2012;26(3):183–8. Epub 2011/10/18. 10.1016/j.trre.2011.07.004 . 22000658

[9] Bach FH, Robson SC, Winkler H, Ferran C, Stuhlmeier KM, Wrighton CJ, et al Barriers to xenotransplantation. Nature medicine. 1995;1(9):869–73. Epub 1995/09/01. . 7585204 10.1038/nm0995-869

[10] Blusch JH, Patience C, Martin U. Pig endogenous retroviruses and xenotransplantation. Xenotransplantation. 2002;9(4):242–51. Epub 2002/06/13. . 12060460 10.1034/j.1399-3089.2002.01110.x

[11] Le Tissier P, Stoye JP, Takeuchi Y, Patience C, Weiss RA. Two sets of human-tropic pig retrovirus. Nature. 1997;389(6652):681–2. Epub 1997/10/24 21:29. 10.1038/39489 . 9338777

[12] Martin U, Kiessig V, Blusch JH, Haverich A, von der Helm K, Herden T, et al Expression of pig endogenous retrovirus by primary porcine endothelial cells and infection of human cells. Lancet. 1998;352(9129):692–4. Epub 1998/09/05. 10.1016/S0140-6736(98)07144-X . 9728985

[13] Scheef G, Fischer N, Krach U, Tonjes RR. The number of a U3 repeat box acting as an enhancer in long terminal repeats of polytropic replication-competent porcine endogenous retroviruses dynamically fluctuates during serial virus passages in human cells. Journal of virology. 2001;75(15):6933–40. Epub 2001/07/04. 10.1128/JVI.75.15.6933-6940.2001 11435573 PMC114421

[14] Takeuchi Y, Patience C, Magre S, Weiss RA, Banerjee PT, Le Tissier P, et al Host range and interference studies of three classes of pig endogenous retrovirus. Journal of virology. 1998;72(12):9986–91. Epub 1998/11/13. 9811736 10.1128/jvi.72.12.9986-9991.1998 PMC110514

[15] Wilson CA, Wong S, Muller J, Davidson CE, Rose TM, Burd P. Type C retrovirus released from porcine primary peripheral blood mononuclear cells infects human cells. Journal of virology. 1998;72(4):3082–7. Epub 1998/04/03. 9525633 10.1128/jvi.72.4.3082-3087.1998 PMC109758

[16] Di Nicuolo G, van de Kerkhove MP, Hoekstra R, Beld MG, Amoroso P, Battisti S, et al No evidence of in vitro and in vivo porcine endogenous retrovirus infection after plasmapheresis through the AMC-bioartificial liver. Xenotransplantation. 2005;12(4):286–92. Epub 2005/06/10. 10.1111/j.1399-3089.2005.00226.x . 15943777

[17] Hermida-Prieto M, Domenech N, Moscoso I, Diaz T, Ishii J, Salomon DR, et al Lack of cross-species transmission of porcine endogenous retrovirus (PERV) to transplant recipients and abattoir workers in contact with pigs. Transplantation. 2007;84(4):548–50. Epub 2007/08/24. 10.1097/01.tp.0000275203.91841.23 . 17713442

[18] Paradis K, Langford G, Long Z, Heneine W, Sandstrom P, Switzer WM, et al Search for cross-species transmission of porcine endogenous retrovirus in patients treated with living pig tissue. The XEN 111 Study Group. Science. 1999;285(5431):1236–41. Epub 1999/08/24. . 10455044 10.1126/science.285.5431.1236

[19] Popp SK, Mann DA, Milburn PJ, Gibbs AJ, McCullagh PJ, Wilson JD, et al Transient transmission of porcine endogenous retrovirus to fetal lambs after pig islet tissue xenotransplantation. Immunology and cell biology. 2007;85(3):238–48. Epub 2007/01/18. 10.1038/sj.icb.7100028 . 17228325

[20] Deng YM, Tuch BE, Rawlinson WD. Transmission of porcine endogenous retroviruses in severe combined immunodeficient mice xenotransplanted with fetal porcine pancreatic cells. Transplantation. 2000;70(7):1010–6. Epub 2000/10/25. . 11045635 10.1097/00007890-200010150-00004

[21] van der Laan LJ, Lockey C, Griffeth BC, Frasier FS, Wilson CA, Onions DE, et al Infection by porcine endogenous retrovirus after islet xenotransplantation in SCID mice. Nature. 2000;407(6800):90–4. Epub 2000/09/19. 10.1038/35024089 . 10993079

[22] Martina Y, Marcucci KT, Cherqui S, Szabo A, Drysdale T, Srinivisan U, et al Mice transgenic for a human porcine endogenous retrovirus receptor are susceptible to productive viral infection. Journal of virology. 2006;80(7):3135–46. Epub 2006/03/16. 10.1128/JVI.80.7.3135-3146.2006 16537582 PMC1440412

[23] Karlas A, Irgang M, Votteler J, Specke V, Ozel M, Kurth R, et al Characterisation of a human cell-adapted porcine endogenous retrovirus PERV-A/C. Annals of transplantation. 2010;15(2):45–54. Epub 2010/07/27. . 20657519

[24] Kim NY, Lee D, Lee J, Park EW, Jung WW, Yang JM, et al Characterization of the replication-competent porcine endogenous retrovirus class B molecular clone originated from Korean domestic pig. Virus genes. 2009;39(2):210–6. Epub 2009/06/23. 10.1007/s11262-009-0377-7 . 19543822

[25] Zmuda EJ, Powell CA, Hai T. A method for murine islet isolation and subcapsular kidney transplantation. Journal of visualized experiments: JoVE. 2011;(50). Epub 2011/04/29. 10.3791/2096 21525838 PMC3169267

[26] Bertera S, Balamurugan AN, Bottino R, He J, Trucco M. Increased yield and improved transplantation outcome of mouse islets with bovine serum albumin. Journal of transplantation. 2012;2012:856386 Epub 2013/01/11. 10.1155/2012/856386 23304445 PMC3523609

[27] Lee JE, Kim G-W, Kim YB, Park HY. Construction of the Porcine Endogenous Retrovirus Envelope Glycoprotein A and B Specific Antibody. Journal of Bacteriology and Virology 2009;39(2):137–43

[28] Atluri VS, Hidalgo M, Samikkannu T, Kurapati KR, Jayant RD, Sagar V, et al Effect of human immunodeficiency virus on blood-brain barrier integrity and function: an update. Frontiers in cellular neuroscience. 2015;9:212 Epub 2015/06/27. 10.3389/fncel.2015.00212 26113810 PMC4461820

[29] Bertin J, Jalaguier P, Barat C, Roy MA, Tremblay MJ. Exposure of human astrocytes to leukotriene C4 promotes a CX3CL1/fractalkine-mediated transmigration of HIV-1-infected CD4(+) T cells across an in vitro blood-brain barrier model. Virology. 2014;454–455:128–38. Epub 2014/04/15. 10.1016/j.virol.2014.02.007 . 24725939

[30] Yang YG, Wood JC, Lan P, Wilkinson RA, Sykes M, Fishman JA, et al Mouse retrovirus mediates porcine endogenous retrovirus transmission into human cells in long-term human-porcine chimeric mice. The Journal of clinical investigation. 2004;114(5):695–700. Epub 2004/09/03. 10.1172/JCI21946 15343388 PMC514590

[31] Martina Y, Kurian S, Cherqui S, Evanoff G, Wilson C, Salomon DR. Pseudotyping of porcine endogenous retrovirus by xenotropic murine leukemia virus in a pig islet xenotransplantation model. American journal of transplantation: official journal of the American Society of Transplantation and the American Society of Transplant Surgeons. 2005;5(8):1837–47. Epub 2005/07/06. 10.1111/j.1600-6143.2005.00978.x . 15996230

[32] Mendoza R, Vaughan AE, Miller AD. The left half of the XMRV retrovirus is present in an endogenous retrovirus of NIH/3T3 Swiss mouse cells. Journal of virology. 2011;85(17):9247–8. Epub 2011/06/24. 10.1128/JVI.05137-11 21697491 PMC3165791

[33] Pal N, Baker R, Schalk S, Scobie L, Tucker AW, Opriessnig T. Detection of porcine endogenous retrovirus (PERV) viremia in diseased versus healthy US pigs by qualitative and quantitative real-time RT-PCR. Transboundary and emerging diseases. 2011;58(4):344–51. Epub 2011/03/15. 10.1111/j.1865-1682.2011.01210.x . 21396084

[34] Sakuma K, Nakao R, Aoi W, Inashima S, Fujikawa T, Hirata M, et al Cyclosporin A treatment upregulates Id1 and Smad3 expression and delays skeletal muscle regeneration. Acta neuropathologica. 2005;110(3):269–80. Epub 2005/06/30. 10.1007/s00401-005-1049-x . 15986223

[35] Koch KS, Son KH, Maehr R, Pellicciotta I, Ploegh HL, Zanetti M, et al Immune-privileged embryonic Swiss mouse STO and STO cell-derived progenitor cells: major histocompatibility complex and cell differentiation antigen expression patterns resemble those of human embryonic stem cell lines. Immunology. 2006;119(1):98–115. Epub 2006/07/14. 10.1111/j.1365-2567.2006.02412.x 16836618 PMC1782333

