# Supplementary material for: Transmission of Porcine Endogenous Retrovirus Produced from Different Recipient Cells In Vivo
Source: PLoS One. 2016 Nov 10;11(11):e0165156. doi: 10.1371/journal.pone.0165156 (PMC5104465; doi:10.1371/journal.pone.0165156)
Supplement: S1 Fig — The genomic DNA was isolated from 293T cells treated with serum from NOG mice transplanted with PERV/NIH3T3 cells. Nested PCR product using pol1, pol2, pol3, and pol4 as primers was cloned to pGEM-T Easy vector and the pol sequence was compared with PERV molecular clone B (EU23109). (PPTX) [file pone.0165156.s002.pptx]

## Slide 1
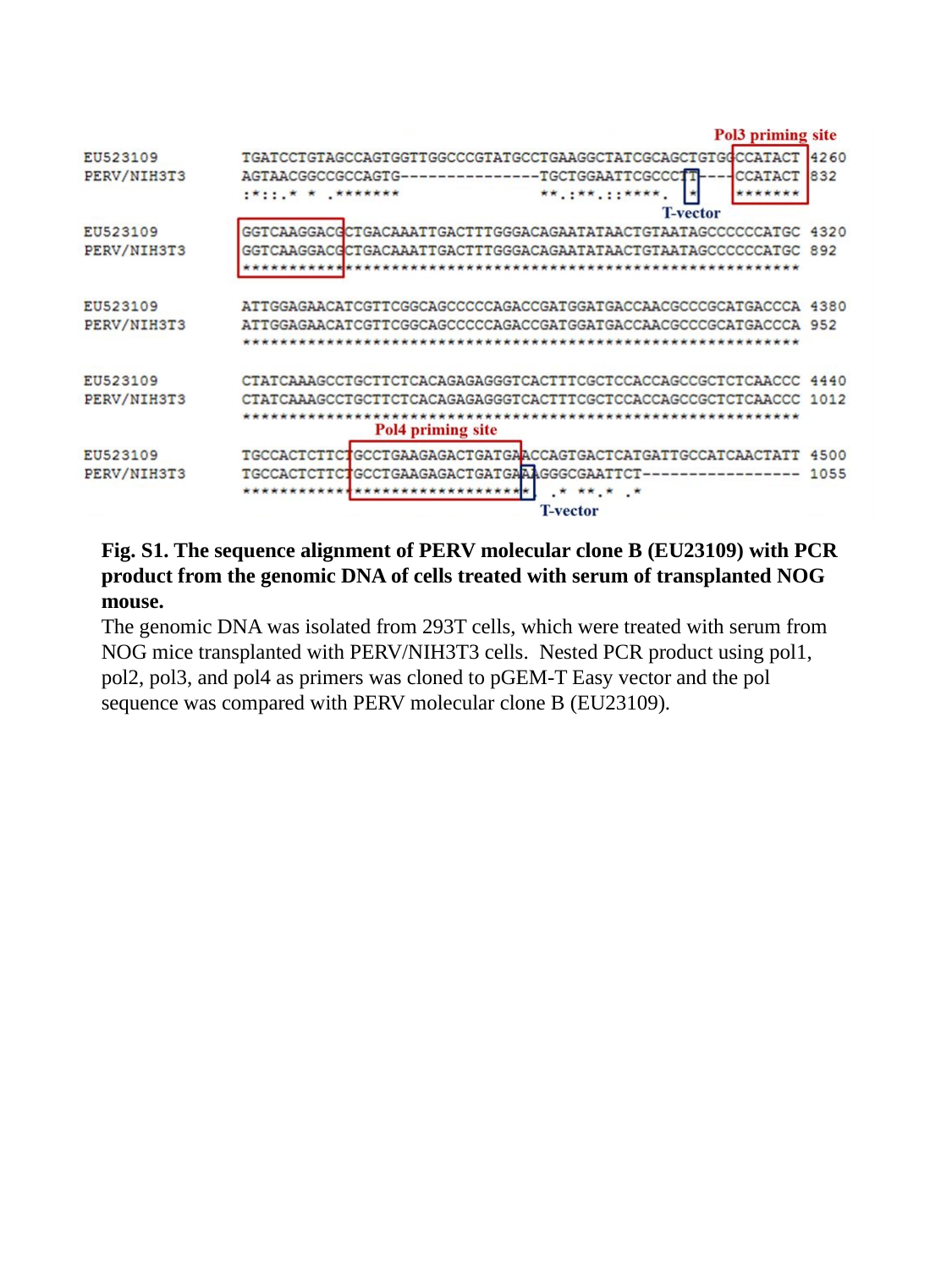

Fig. S1. The sequence alignment of PERV molecular clone B (EU23109) with PCR product from the genomic DNA of cells treated with serum of transplanted NOG mouse.
The genomic DNA was isolated from 293T cells, which were treated with serum from NOG mice transplanted with PERV/NIH3T3 cells. Nested PCR product using pol1, pol2, pol3, and pol4 as primers was cloned to pGEM-T Easy vector and the pol sequence was compared with PERV molecular clone B (EU23109).
