# Supplementary material for: Transmission of Porcine Endogenous Retrovirus Produced from Different Recipient Cells In Vivo
Source: PLoS One. 2016 Nov 10;11(11):e0165156. doi: 10.1371/journal.pone.0165156 (PMC5104465; doi:10.1371/journal.pone.0165156)
Supplement: S2 Fig — (A) The expected annealing location of primers (arrows) was illustrated in the genomes of PERV and mERV-XL (32). (B) The PCR products of PERV gag-pol and mERV-XL gag-pol with the listed primers were detected in lanes 1, 6 and lanes 2, 3, respectively. The PCR product of a possible recombinant PERV with mERV-XL was not detected in lane 4,5,7,8. (C) Nucleotide sequence of the primers used for the PCR in (B) was listed. (PPTX) [file pone.0165156.s003.pptx]

## Slide 1
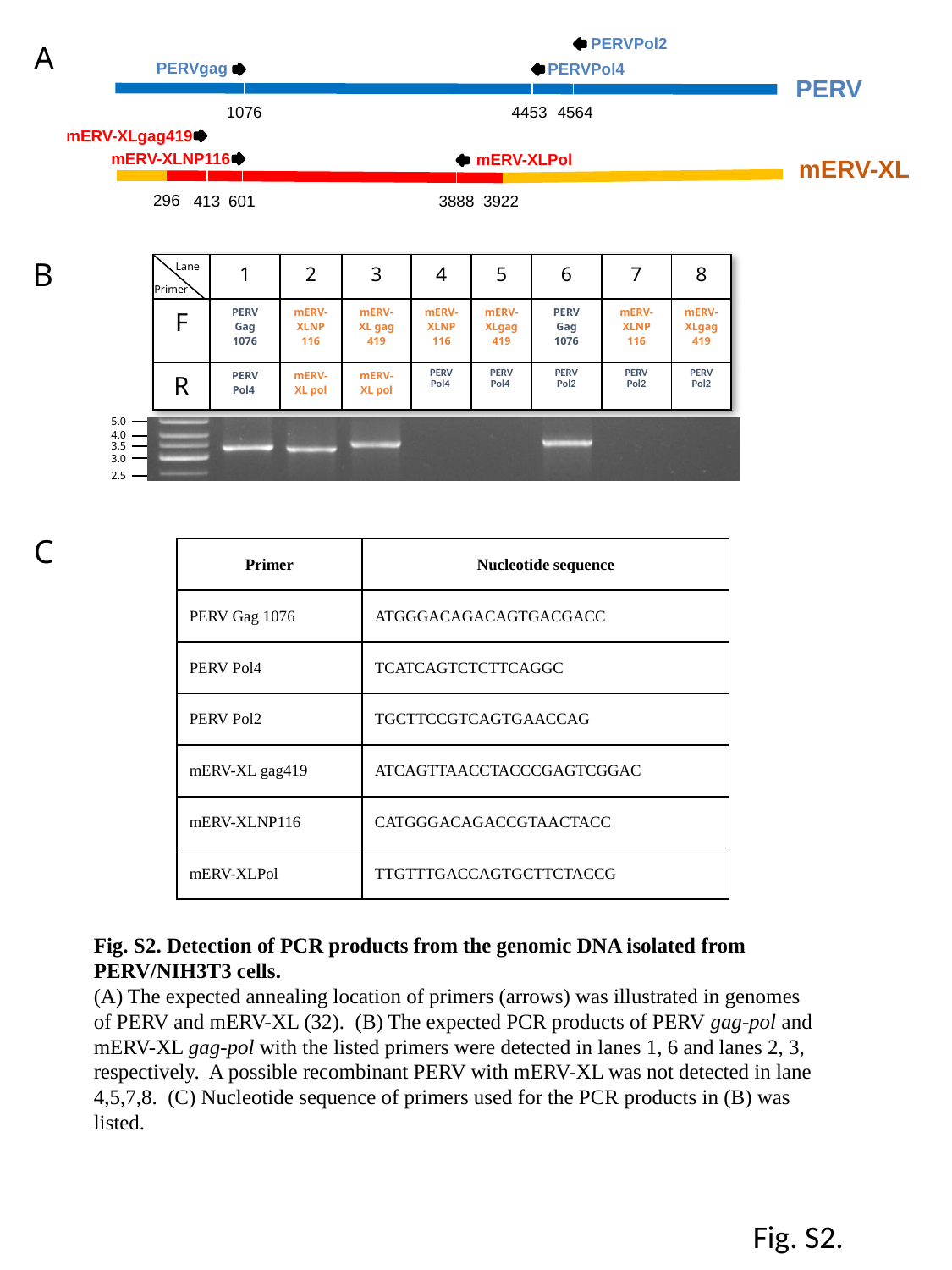

PERVPol2
A
PERVgag
PERVPol4
PERV
1076
4453
4564
mERV-XLgag419
mERV-XLNP116
mERV-XLPol
mERV-XL
296
413
601
3888
3922
B
Lane
| | 1 | 2 | 3 | 4 | 5 | 6 | 7 | 8 |
| --- | --- | --- | --- | --- | --- | --- | --- | --- |
| F | PERV Gag 1076 | mERV-XLNP 116 | mERV-XL gag 419 | mERV-XLNP 116 | mERV-XLgag419 | PERV Gag 1076 | mERV-XLNP 116 | mERV-XLgag419 |
| R | PERV Pol4 | mERV-XL pol | mERV-XL pol | PERV Pol4 | PERV Pol4 | PERV Pol2 | PERV Pol2 | PERV Pol2 |
Primer
5.0
4.0
3.5
3.0
2.5
C
| Primer | Nucleotide sequence |
| --- | --- |
| PERV Gag 1076 | ATGGGACAGACAGTGACGACC |
| PERV Pol4 | TCATCAGTCTCTTCAGGC |
| PERV Pol2 | TGCTTCCGTCAGTGAACCAG |
| mERV-XL gag419 | ATCAGTTAACCTACCCGAGTCGGAC |
| mERV-XLNP116 | CATGGGACAGACCGTAACTACC |
| mERV-XLPol | TTGTTTGACCAGTGCTTCTACCG |
Fig. S2. Detection of PCR products from the genomic DNA isolated from PERV/NIH3T3 cells.
(A) The expected annealing location of primers (arrows) was illustrated in genomes of PERV and mERV-XL (32). (B) The expected PCR products of PERV gag-pol and mERV-XL gag-pol with the listed primers were detected in lanes 1, 6 and lanes 2, 3, respectively. A possible recombinant PERV with mERV-XL was not detected in lane 4,5,7,8. (C) Nucleotide sequence of primers used for the PCR products in (B) was listed.
Fig. S2.
